# Acute worsening of native lung fibrosis after single lung transplantation for pulmonary fibrosis: two case reports

**DOI:** 10.1186/s13256-021-03191-9

**Published:** 2022-01-03

**Authors:** Tiphaine Goletto, Sixtine Decaux, Vincent Bunel, Gaëlle Weisenburger, Jonathan Messika, Samer Najem, Chahine Medraoui, Cendrine Godet, Marie Pierre Debray, Brice Lortat-Jacob, Pierre Mordant, Yves Castier, Lila Bouadma, Raphael Borie, Hervé Mal

**Affiliations:** 1 Service de Pneumologie B, Hôpital Bichat, Assistance Publique-Hôpitaux de Paris, 75018 Paris, France; 2 Inserm UMR1152, Université Paris7 Denis Diderot, 75018 Paris, France; 3 Service de Radiologie, Hôpital Bichat, Paris, France; 4 Service de Chirurgie Vasculaire, Thoracique et Transplantation Pulmonaire, Hôpital Bichat, Paris, France; 5 Service de Réanimation Chirurgicale, Hôpital Bichat, Paris, France; 6 Service de Réanimation Médicale et Infectieuse, Hôpital Bichat, Paris, France; 7 Service de Pneumologie A, Hôpital Bichat, Paris, France; 8 Service de Pneumologie B et Transplantation Pulmonaire, Hôpital Bichat, 46 rue Henri Huchard, 75018 Paris, France

**Keywords:** Lung fibrosis, Idiopathic pulmonary fibrosis, Hypoxemia, Lung infection, Lung transplantation

## Abstract

**Background:**

In patients receiving single lung transplantation for idiopathic pulmonary fibrosis, worsening of fibrosis of the native lung is usually progressive over time, with no significant effects on gas exchange.

**Case presentation:**

Here, we describe the cases of two Caucasian male recipients of single lung transplants for idiopathic pulmonary fibrosis, 65 and 62 years of age, who exhibited acute worsening of lung fibrosis after an episode of serious viral infection (cytomegalovirus primo-infection in one case and COVID-19 in the other). In both cases, along with opacification of the native lung over several days, the patients presented acute respiratory failure that required the use of high-flow nasal oxygen therapy. Eventually, hypoxemic respiratory failure resolved, but with rapid progression of fibrosis of the native lung.

**Conclusion:**

We conclude that acute worsening of fibrosis on the native lung secondary to a severe viral infection should be added to the list of potential complications developing on the native lung after single lung transplantation for idiopathic pulmonary fibrosis.

## Background

Single lung transplantation (SLT) and bilateral lung transplantation are validated therapeutic options for managing advanced forms of chronic respiratory failure. The choice of the technique depends on the age, general condition, and lung pathology of the recipient. Worldwide, bilateral lung transplantation is now the most frequently used lung transplantation technique [1], but SLT, which is technically simpler, may be a reasonable option for patients with chronic obstructive pulmonary disease or lung fibrosis who are ≥ 60–65 years old and/or present frailty or comorbid conditions [2]. However, one major drawback of SLT is that recipients are exposed to complications involving the native lung [3, 4], such as infection, hemoptysis, pneumothorax, and neoplasia. In patients receiving SLT for idiopathic pulmonary fibrosis (IPF), fibrosis of the native lung usually worsens over time [5–8], with no significant effects on gas exchange [9]. Here, we describe two recipients of SLT for IPF who exhibited acute worsening of lung fibrosis after an episode of serious viral infection: cytomegalovirus primo-infection in one case and COVID 19 in the other. Before transplantation, the patients gave their consent to the anonymous utilization of their pre- and postoperative data.

## Case presentation

### Case 1

A 65-year-old Caucasian man underwent right SLT in January 2020 for IPF. Cytomegalovirus (CMV) mismatch (positive donor, negative recipient) required valganciclovir prophylaxis. Lung perfusion scan performed 2 months after transplantation showed residual perfusion toward the native lung, at 43% of the total perfusion.

On quantitative PCR, the CMV DNA viral load became detectable in the blood (DNAemia) in April 2020, and was treated with therapeutic doses of valganciclovir. Despite this management, the viral load increased in May 2020, and intravenous CMV immunoglobulin was added to the therapeutic regimen.

**Fig. 1:**
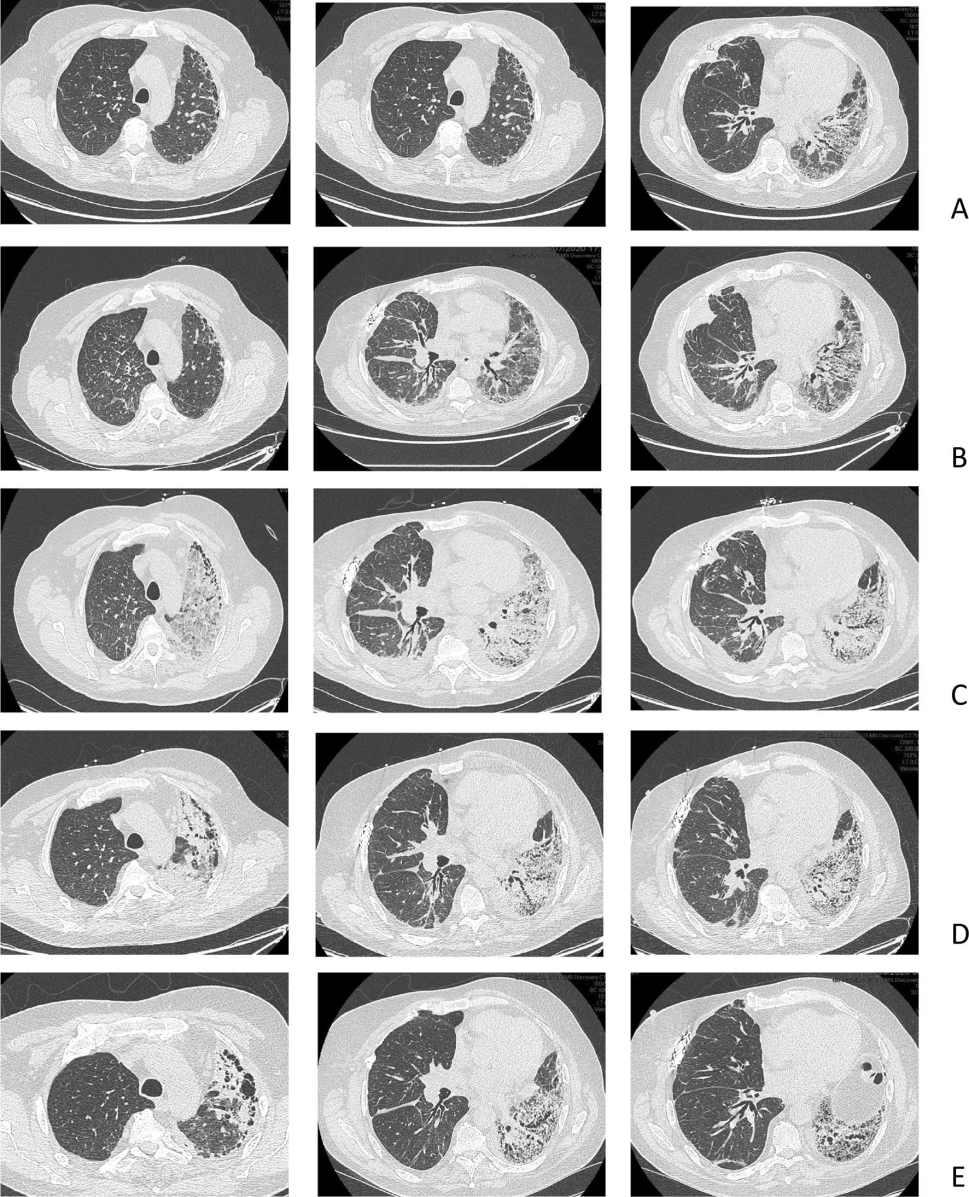
CT of the thorax in patient 1, a 65-year-old man, at various times after right single lung transplantation (SLT) for idiopathic pulmonary fibrosis (IPF). **A** Day 7 after hospital admission (day 1) for management of CMV infection in a patient with CMV mismatch. **B** Day 14, 1 day before the patient developed acute respiratory failure (ARF) requiring a transfer to the intensive care unit (ICU) on day 16. **C** Day 19, while in the ICU for ARF. **D** Day 27, 1 day before discharge from the ICU. **E** Day 63, well after the ARF episode.

The patient was hospitalized in July 2020 (day 1) with persistent CMV viral load, with no clinical arguments for CMV disease. The persistent viral load (with peak load of 411,303 copies) despite the use of intravenous ganciclovir initiated on admission prompted the search for CMV resistance (CMV genotype test), which revealed resistance to gangiclovir (UL97 genotype). Treatment with intravenous foscarnet was initiated on day 13, along with hydration to prevent acute deterioration of the renal function. Results of thoracic computed tomography (CT) scan performed on day 7 are shown in Fig. 1A.

On day 15, the respiratory status of the patient worsened, with fever, cough, and acute respiratory failure, requiring transfer to the intensive care unit (ICU) on day 16. On chest CT, mild pleural effusion and interlobular septal thickening in the lower part of the pulmonary field of the right grafted lung were the only imaging manifestations on day 14 (Fig. 1B). Bronchoalveolar lavage (BAL) results were negative for bacterial, fungal, or viral pathogens, except for CMV with a positive PCR result. Chest CT on day 19 (Fig. 1C) showed a densification of the native lung as compared with the CT performed 5 days earlier with ground glass opacities of the left apex. The underlying mechanism of the acute respiratory failure was suspected to be pulmonary edema secondary to fluid overload associated with foscarnet infusion.

Despite large-spectrum antibiotic therapy and volume depletion, oxygen requirement increased (6 L/min on admission to the ICU, introduction of high-flow nasal oxygen therapy on day 17, up to 40 L/min, FiO_2_ 80% on day 20). Results of BAL, repeated on day 27, were negative for bacterial or fungal pathogens, and the CMV viral load on PCR was decreased. A chest CT performed on day 27 confirmed the marked densification of the native lung, along with the disappearance of the CT abnormalities on the right lung (Fig. 1D). From day 26, the respiratory condition improved progressively, which allowed for discharge from the ICU to the general ward on day 28, with persistent requirement for oxygen therapy at rest (4 L/min). Meanwhile, CMV DNAemia decreased under foscarnet infusion.

From day 28 to final discharge from the hospital 45 days later, the patient showed slow respiratory improvement. Oxygen saturation at rest was 97% at the time of discharge from the hospital. When PCR revealed no CMV viral load, foscarnet was replaced with letermovir.

A chest CT performed well after the acute respiratory failure (day 63) showed a persistent densification of the native lung predominating on the lower field (Fig. 1E). At no time during the course of the treatment was native lung biopsy considered because of the risks of morbidity/mortality of the procedure and the absence of an expected benefit for the patient.

### Case 2

**Fig. 2:**
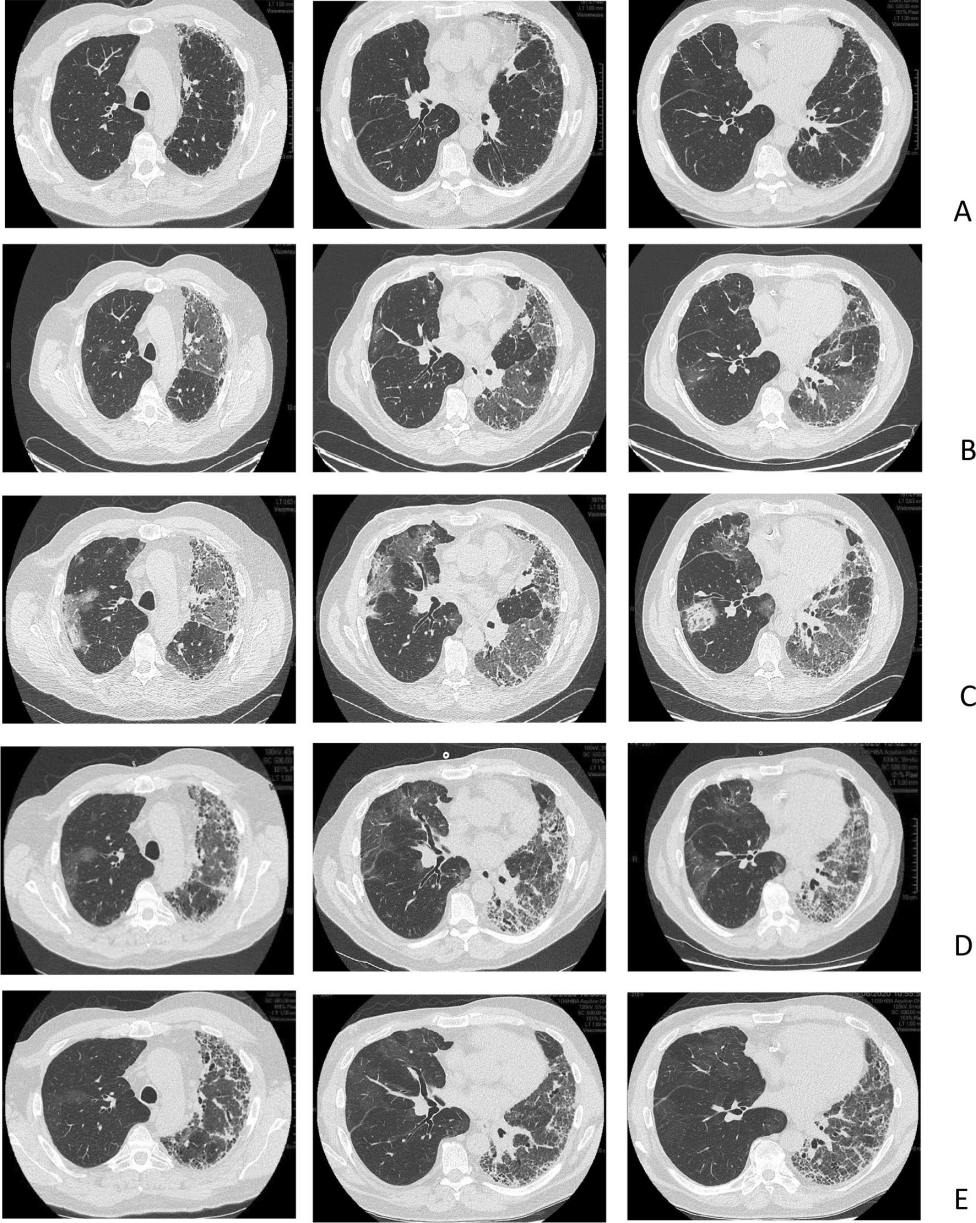
CT of the thorax in patient 2, a 62-year-old man, at different times after right SLT for IPF. **A** Postoperative day (POD) 90, before SARS-CoV-2 infection. **B** POD 104, when SARS-CoV-2 infection was diagnosed. **C** POD 110, at the time of transfer to the ICU for ARF. **D** POD 135, after discharge from the ICU. **E** POD 170 after discharge from the hospital.

In December 2019, a 62-year-old Caucasian man underwent right SLT for IPF. The early postoperative course was uneventful except for two episodes of acute cellular rejection on postoperative days (PODs) 40 and 90 that were treated with a bolus of methylprednisolone, which returned the FEV1 to the prerejection value. On lung perfusion scan performed on POD 60, 35% of the total lung perfusion was directed toward the left native lung. Chest CT results on POD 90 are shown in Fig. 2A.

In April 2020, the patient presented cough and decreased FEV1. Chest CT on POD 104 showed bilateral ground glass opacities with native lung predominance (Fig. 2B). PCR nasopharyngeal swab results positive for SARS-CoV-2 confirmed the diagnosis of COVID-19.

The respiratory condition worsened over the following week and required oxygen therapy. Despite the introduction of lopinavir/ritonavir followed by remdesivir, breathlessness and oxygen requirement increased, so the patient was transferred to the ICU, where high-flow nasal oxygen therapy was initiated. Thoracic CT angiography performed on POD 110 showed worsening of the ground glass opacities (still with native lung predominance) (Fig. 2C). BAL results were negative for bacterial, fungal, and viral pathogens (except for SARS-CoV-2).

After several days under high-flow nasal oxygen therapy (up to 40 L/min, 50% FiO_2_), the respiratory condition of the patient improved, which allowed for discharge from the ICU to the general ward on day 115 under 5 L/min nasal oxygen therapy. The improvement was slow over the following weeks, with a progressive decrease in oxygen requirement. On POD 135, thoracic CT revealed a regression of the ground glass opacities on the grafted side and a marked progression of pulmonary fibrosis on the native lung (Fig. 2D). Lung perfusion scan performed on day 126 showed perfusion in the left lung representing 11% of the total perfusion. On the day of discharge from hospital (POD 155), the patient was weaned from oxygen at rest but needed oxygen upon exercise. PaO_2_ and PaCO_2_ values on that day, with room air at rest, were 63 and 38 mmHg, respectively. Thoracic CT performed on POD 170 revealed a normal appearance of lung parenchyma on the grafted side and marked fibrotic lesions on the native lung (Fig. 2E).

## Discussion

After SLT, the native lung may be the target of possible complications, which may be a source of morbidity or mortality [3, 10, 11]. If the underlying pathology is pulmonary fibrosis, the native lung is exposed to infection, including mycobacterial and fungal infection, pneumothorax, hemoptysis, neoplasia in the form of bronchopulmonary cancer, or lymphoproliferative disease. In general, lung fibrosis affecting the remaining lung is not by itself directly responsible for significant morbidity. The usual profile is that of a progressive worsening of lung fibrosis over time. In 21 patients who underwent SLT for IPF and were followed for an average of 35 months after transplant, sequential high-resolution chest CT revealed a progressive loss of lung volume and extension of the fibrotic lesions over time in the native lung [12]. Similarly, in smaller studies retrospectively analyzing 5–13 SLT patients by use of a chest CT scoring system, other investigators described progressive worsening of fibrosis (and decrease in lung volume) in the native lung [5, 6, 8]. In contrast with the usual profile of progressive worsening of lung fibrosis, acute worsening of pulmonary fibrosis in the native lung seems to be much less frequent. The Vancouver group reported a single observation: 4 years after SLT for IPF, the patient presented acute respiratory symptoms, the chest CT revealing ground glass opacities throughout the native lung, which were not present 3 months earlier, without recent abnormalities in the grafted lung [13]. The diagnostic workup was negative, including microbiology analysis. Within 10 days, the native lung showed progressive opacification, which led to considering the diagnosis of acute exacerbation of IPF and to administer prednisone 1 mg/kg along with *N*-acetylcysteine, with subsequent improvement of the native lung on high-resolution chest CT. Despite the absence of pathology confirmation, this observation is consistent with the diagnosis of acute exacerbation of IPF on the remaining lung. Recently, Marron *et al*. described three IPF patients who experienced acute worsening of pulmonary fibrosis in the native lung after SLT [14]. All three patients were seronegative for CMV and received a graft from a seropositive donor. More than 12 months after SLT, in a context of CMV primo-infection with positive results of CMV DNA quantitative PCR in the serum, the patients presented acute hypoxemic respiratory failure. There were no radiographic or chest CT signs of CMV pneumonia affecting the graft, but within several days, opacification of the native lung was observed. The patients received CMV curative therapy, and eventually hypoxemic respiratory failure resolved, but with rapid progression of fibrosis of the native lung.

Our two patients share several similarities with those described by Marron *et al*. First, in both reports, patients showed rapid opacification (within several days) of the native lung. Whether the patients experienced acute exacerbation of IPF or simply rapid progression of pulmonary fibrosis is not clear from the reports by Marron *et al*. Chest CT in both of our patients revealed a pattern of ground glass opacities superimposed on pulmonary fibrosis, which is consistent with the diagnosis of acute exacerbation of IPF despite the absence of histological data.

A second point common to both reports is that patients presented hypoxemic respiratory failure, along with the opacification of the native lung. In our case of CMV primo-infection, as in the three previously published cases, CMV DNA replication was detected in serum, but signs suggesting CMV pneumonia, known to mainly involve the lung allograft in case of SLT [4], were missing on chest radiographs or chest CT. This observation raises the question of the mechanism of the hypoxemic respiratory failure presented by the patients. The severity of the parenchymal involvement of the native lung may have led to major ventilation/perfusion mismatch, with the progressive improvement of the respiratory condition mainly related to the subsequent decrease of the perfusion toward the native lung over time, rather than to antiviral treatment. Finally, both reports have in common a rapid progression of pulmonary fibrosis in a context of severe viral infection, which in our report was CMV primo-infection in one patient and COVID-19 in the other. The mechanism leading to the rapid progression of fibrosis may not be specific to CMV, but rather due to the direct viral aggression of an already injured parenchyma.

## Conclusion

In conclusion, acute worsening of fibrosis on the native lung secondary to a severe viral infection should be added to the list of potential complications developing on the native lung after SLT for pulmonary fibrosis.

## Data Availability

The data we present are available in our lung department in the form of electronic files (clinical, biological and imaging data).

## Abbreviations

SLT: Single lung transplantation
IPF: Idiopathic pulmonary fibrosis
CMV: Cytomegalovirus
DNAemia: CMV DNA viral load in the blood
ICU: Intensive care unit
BAL: Bronchoalveolar lavage
POD: Postoperative day
